# Fructose Promotes Oxidative Stress, Sphingolipid Remodelling and Transient Intestinal Barrier Dysfunction in Differentiated Caco-2 Cells

**DOI:** 10.3390/nu18203359

**Published:** 2026-10-14

**Authors:** Gabriele Serreli, Maria Paola Melis, Claudia Guerriero, Cristina Manis, Giulia Tolle, Giuseppina Sanna, Germano Orrù, Sara Fais, Pierluigi Caboni, Monica Deiana

**Affiliations:** 1 Department of Biomedical Sciences, University of Cagliari, 09042 Monserrato, Italy; 2 Department of Life and Environmental Sciences, University of Cagliari, 09042 Monserrato, Italy; 3 Oral Biotechnology Laboratory (OBL), Department of Surgical Sciences, University of Cagliari, 09121 Cagliari, Italy

**Keywords:** Caco-2 cells, ceramides, fructose, intestinal barrier, oxidative stress, sphingolipid metabolism, tight junctions

## Abstract

**Background/Objectives**: High dietary fructose consumption has been linked to impaired intestinal barrier function and several metabolic disorders. Although oxidative stress has been proposed as a mediator of fructose-related epithelial injury, the involvement of tight-junction (TJ) proteins and sphingolipid metabolism is still not fully defined. Here, differentiated Caco-2 monolayers were exposed to fructose (1–50 mM) to examine changes in epithelial barrier integrity and associated molecular responses. **Methods**: Cell viability, transepithelial electrical resistance (TEER), reactive oxygen species (ROS), intracellular reduced and oxidized glutathione (GSH/GSSG) ratio, nitrites as an indirect assessment of nitric oxide (NO) production, TJ protein level (ZO-1, JAM-A and occludin), sphingolipid profile by UHPLC-QTOF-MS, and expression of the ceramide synthases CERS2 and CERS6 were evaluated. **Results**: Fructose did not affect cell viability but induced a rapid, concentration-dependent increase in ROS generation, an early reduction in the GSH/GSSG ratio, and increased nitrites production. The observed alterations were concurrent with a temporary reduction in TEER, which returned to control levels at subsequent time points. Fructose selectively reduced JAM-A and ZO-1 protein expression, whereas occludin remained unaffected. Lipidomic analysis revealed sphingolipid remodelling, characterised by increased ceramide species and reduced sphingomyelins, while gene expression analysis showed downregulation of CERS2, with CERS6 remaining unchanged. The altered sphingolipid profile mainly involved long- and very-long-chain ceramides, suggesting a possible link between altered membrane lipid homeostasis and barrier dysfunction. **Conclusions**: Fructose exposure transiently compromises intestinal epithelial barrier function and is associated with oxidative stress, changes in TJ proteins, and sphingolipid remodelling. These preliminary findings identify sphingolipid remodelling as a candidate contributor to fructose-induced intestinal barrier dysfunction, warranting confirmation through dedicated mechanistic follow-up studies.

## 1. Introduction

Fructose is a monosaccharide with strong lipogenic potential that occurs naturally in fruits and several vegetables. In the diet, however, substantial amounts are provided by sucrose and high-fructose corn syrup, which are widely used in sweetened beverages and processed foods. Among dietary sources of added fructose, sugar-sweetened beverages account for a major proportion of intake in Western populations, especially in children and adolescents [1,2]. High fructose consumption has been linked to metabolic syndrome, visceral adiposity, insulin resistance and non-alcoholic fatty liver disease (NAFLD/MASLD), through activation of hepatic de novo lipogenesis and suppression of fatty acid oxidation [3,4]. Its metabolic peculiarity and the association with metabolic dysfunction combined with its ubiquity in the current food system, have led some authors to describe fructose as a genuine “metabolic toxin” [5], whose deleterious action is not confined to the liver but, as increasingly recognised, also extends to the intestine, that is the primary site of its absorption and first-pass metabolism.

Evidence from preclinical models indicates that high fructose exposure can modify gut microbiota composition, increase intestinal permeability and favour systemic endotoxaemia [6,7], in part through reduced production of gut-barrier proteins that limit bacterial and endotoxin translocation [4]. At the epithelial level, these effects have been associated with oxidative and nitrosative stress [8], which damage tight-junction (TJ) proteins (ZO-1, occludin, claudins) and the adherens-junction component JAM-A via their delocalisation and degradation [9,10], accompanied by alterations in the intracellular GSH/GSSG ratio, a hallmark of cellular redox status [11].

A further, less explored layer of complexity concerns membrane lipid remodelling. Ceramides are bioactive sphingolipids produced predominantly through de novo synthesis from palmitoyl-CoA and serine and can influence intestinal barrier integrity in an isoform-dependent manner: in the colon of ceramide synthase 2 (CERS2) knockout mice, JAM-A expression is markedly decreased and CERS2 deficiency worsens experimental colitis, implicating this isoform in inflammatory bowel disease (IBD) pathogenesis [12]. The six CERS isoforms differ in the ceramide species they preferentially generate, resulting in distinct acyl-chain profiles and biological functions, with CERS2 synthesising barrier-essential very-long-chain ceramides and CERS6 producing C16-ceramides linked to lipotoxicity and pro-inflammatory signalling [12,13]. Notably, this relationship is not unidirectional: besides insufficient very-long-chain ceramide synthesis, an excess of long- and very-long-chain ceramide species above physiological levels can itself destabilise membrane organisation, increasing lipid packing order and promoting ceramide-enriched gel domains [14,15]. Intestinal barrier integrity may therefore depend on a tightly regulated ceramide “set-point” rather than on the abundance of a single species.

Ceramide metabolism and cellular redox balance are closely interconnected in a bidirectional manner. Increased ROS can promote ceramide accumulation by stimulating ceramide-producing pathways and favouring the ceramide/S1P balance toward ceramide [16,17], including through direct activation of sphingomyelinases—most remarkably acid and neutral sphingomyelinase (aSMase and nSMase, respectively)—independently of de novo biosynthesis; in epithelial cells, H_2_O_2_ and reactive nitrogen species (RNS) have been shown to engage distinct sphingomyelinase isoforms to generate ceramides [18,19]. Conversely, elevated ceramide levels may further enhance ROS formation through mechanisms involving mitochondrial dysfunction, NADPH oxidase activity and signalling from ceramide-enriched membrane domains, a feedforward loop reported across vascular, renal and inflammatory contexts [16,17,18,20]. This axis is also nutritionally modulated: diets rich in saturated fat and sugar raise specific long-chain ceramide species [21], and large-scale epidemiological data from the Framingham Offspring Cohort directly link chronic high fructose consumption, mainly via sugar-sweetened beverages, to elevated circulating deleterious ceramides (Cer(d18:1/16:0), Cer(d18:1/22:0) and Cer(d18:1/24:0)) particularly in individuals with prediabetes or type 2 diabetes [22].

Taken together, these observations suggest a potential connection among fructose exposure, oxidative stress, junctional protein alterations, sphingolipid metabolism and inflammation, which may contribute to intestinal barrier disruption, consistent with the rising IBD incidence observed in countries that have adopted a Western-style dietary pattern [23]. Based on this rationale, we investigated the effects of fructose on intestinal epithelial barrier function using differentiated Caco-2 cells as an in vitro model of the human intestinal epithelium. We evaluated the Caco-2 monolayer integrity, using transepithelial electrical resistance (TEER) as an index of barrier alteration, the abundance of ZO-1, JAM-A and occludin, ROS production, the GSH/GSSG ratio, nitrites levels as an indirect assessment of nitric oxide (NO) production, lipidomic alterations with particular emphasis on ceramides, and CERS2/CERS6 gene expression. The integration of these functional, molecular and lipidomic endpoints was intended to provide a coherent mechanistic picture of how fructose, at nutritionally relevant concentrations, may compromise intestinal barrier homeostasis, contributing to clarify its role as a modifiable environmental risk factor in IBD.

## 2. Materials and Methods

### 2.1. Materials for Cell Culture

Phosphate-buffered saline (PBS) with and without MgCl_2_ and CaCl_2_, fetal bovine serum (FBS), antibiotic penicillin/streptomycin 1× and Dulbecco’s Modified Eagle Medium (DMEM) low glucose with L-arginine, were purchased from Euroclone (Milan, Italy). The low-glucose DMEM used for both cell maintenance/differentiation and all fructose treatments (Euroclone, cat. no. ECM0060L) contains 1000 mg/L (5.5 mM) D-glucose, 584 mg/L L-glutamine and 110 mg/L (1 mM) sodium pyruvate; this basal glucose concentration was therefore constant across all experimental groups, including untreated controls, in every assay described below.

### 2.2. Cell Culture Maintenance

Caco-2 cells, obtained from ECACC (Salisbury, UK), were used as an in vitro model of the intestinal epithelium. Upon reaching confluence, these human colorectal adenocarcinoma cells undergo spontaneous differentiation and acquire an enterocyte-like phenotype. Cells were cultured in T75 flasks until approximately 80% confluence using DMEM supplemented with 10% FBS and 1% antibiotic/antifungal solution (100 U/mL penicillin and 100 mg/mL streptomycin) at 37 °C under 5% CO_2_ [24]. Cells between passages 45 and 60 were detached with 1% trypsin at 37 °C for 5–10 min. Following centrifugation, cell number was determined using a Bürker chamber, and cells were seeded in 6- or 96-well plates, Transwell inserts or Petri dishes at 5 × 10^4^ cells/mL, according to the experimental procedure. Differentiation was allowed to proceed for 14–21 days, with medium replacement performed twice weekly.

### 2.3. Cell Viability Assay

Cell viability following fructose exposure was assessed using the MTT colorimetric assay. Differentiated Caco-2 cells were seeded in 96-well plates before fructose treatment. Fructose (1–50 mM) was added directly to complete growth medium (DMEM low glucose, 10% FBS) for the 24 h incubation; untreated cells (0 mM) received an equal volume of vehicle (medium only). Following treatment, MTT solution was added (5 mg/mL diluted to 8% final concentration in culture medium) for 4 h at 37 °C. After removal of the MTT-containing medium, 100 µL DMSO was added to each well and absorbance was recorded at 570 nm using a Tecan Infinite 200 microplate reader (Infinite 200, Tecan, Salzburg, Austria). Viability data were reported as % of control (untreated cells).

### 2.4. Determination of Intracellular ROS Production

Caco-2 cells were plated in 96-well plates at 5 × 10^4^ cells/mL and maintained until differentiation was complete. After washing with PBS, cells were loaded with 2′-7′-dichlorofluorescein diacetate (H_2_-DCF-DA, Merck, Milan, Italy) for 30 min at 10 μM concentration [25]. The probe-containing solution was subsequently removed and replaced with fructose-containing medium (1–50 mM); control cells (0 mM) were incubated in the same medium without fructose. Following 1 h of fructose exposure, the treatment medium was removed, PBS was added, and fluorescence was recorded at 37 °C. Fluorescence was measured at 490 nm excitation and 520 nm emission. Fluorescence was monitored for 2 h, with measurements collected at 5-min intervals.

### 2.5. Determination of Reduced (GSH) and Oxidized (GSSG) Intracellular Glutathione

Intracellular GSH and GSSG were quantified by HPLC with electrochemical detection (HPLC-EC), following the analytical approach previously reported [26,27]. For this analysis, differentiated Caco-2 cells were cultured in 6-well plates at 1 × 10^5^ cells/2 mL. Fructose (25 and 50 mM) was added to complete growth medium (DMEM low glucose, 10% FBS) for different exposure times (2, 4 and 24 h); control cells were maintained in the same medium without fructose for the corresponding time. At the end of treatment, cells were recovered in 500 µL PBS and centrifuged at 12,500 rpm for 7 min. The resulting pellet was extracted using 150 µL of 10% metaphosphoric acid (MPA) together with 150 µL of 0.05% trifluoroacetic acid (TFA) (Merck, Milan, Italy). After centrifugation, 5 μL were collected for protein determination and the remaining supernatant was injected into the HPLC system. The amounts of GSH and GSSG were measured using an HPLC (Agilent 1260 infinity, Agilent Technologies, Palo Alto, CA, USA) equipped with an electrochemical detector (DECADE II Antec, Leiden, The Netherlands) and an Agilent 35900E interface. Calibration curves were generated from GSH and GSSG standards prepared at different concentrations. The GSH/GSSG ratio was normalised to total protein content and expressed relative to the untreated control, assigned a value of 100%.

### 2.6. Determination of Transepithelial Electrical Resistance (TEER)

Differentiated Caco-2 cells seeded (5 × 10^4^/mL in 500 μL) in Transwell plates (polycarbonate membranes with 0.4 μm pore diameter, Sigma-Aldrich, Milan, Italy) were used to determine TEER as described elsewhere [28]. Briefly, cells in media showing TEER values greater than 300 Ω·cm^2^ were treated with fructose (25–50 mM) for 72 h. TEER was measured every 15 min until 72 h and values expressed as a percentage of TEER at time 0 (considered as 100% TEER).

### 2.7. Determination of Nitrite Levels

Nitrite levels, as an indirect marker of NO production, were assessed in Caco-2 cells cultured in 6-well plates (5 × 10 ^4^ cells/mL in 2 mL) in phenol red-free DMEM with 0.1 mM L-arginine and 2.5% fetal bovine serum. Once differentiated, cells were exposed to fructose (1–50 mM) for 48 h before NO determination. At the end of treatment, culture medium was collected and 100 µL aliquots were mixed 1:1 with Griess reagent (Sigma Aldrich, Milan, Italy) and incubated for 20 min at room temperature [29]. Absorbance was subsequently recorded at 540 nm, and nitrite concentrations were calculated against a sodium nitrite calibration curve (0.1–10 µM). The results were expressed as μmol/L of nitrite secreted by the cells.

### 2.8. Western Blot Analyses

Differentiated Caco-2 cells were cultured in 6-well plates at a density of 5 × 10^4^ cells/mL in DMEM supplemented with 0.1 mM L-arginine and 2.5% fetal bovine serum. Cells were exposed to fructose (10, 25, or 50 mM) for 1 or 3 h. The Western blot procedure was performed based on a previously described protocol [30], with modifications according to the experimental conditions of the present study. At the end of the treatment, the culture medium was removed, and cells were lysed in 180 µL of CellLytic™ M commercial lysis buffer (Sigma-Aldrich, Milan, Italy) supplemented with Pierce™ phosphatase and protease inhibitor mini tablets (Thermo Fisher Scientific, Waltham, MA, USA). Cell lysates were centrifuged at 12,500× *g* for 7 min at 4 °C, and the resulting supernatants were collected. Total protein concentration was determined using the Bradford assay [31]. Samples were either processed immediately or stored at −20 °C until Western blot analysis. For each sample, 40 µg of reduced and denatured proteins were separated by electrophoresis on 10% polyacrylamide gels and subsequently transferred onto nitrocellulose membranes. Membranes were blocked for 30 min at room temperature with 25 mL of TTBS containing 4% dry milk. TTBS consisted of 20 mM Tris/HCl (pH 7.5), 100 mM NaCl, and 0.1% Tween 20.

Membranes were incubated overnight at 4 °C on a three-dimensional rocking platform with primary antibodies against ZO-1 (ab216880), occludin (ab216327), JAM-A (ab52647), and β-actin (ab8227) (Abcam, Cambridge, UK), diluted 1:1000 in TTBS containing 1% dry milk. All primary antibodies used in this study are commercially validated for specificity by the manufacturer (Abcam) and have been previously employed to detect the corresponding TJ and adherens-junction proteins in intestinal epithelial cell models, including our own previous works [28,32]. After two washes with TTBS, membranes were incubated for 45 min at room temperature with either HRP-conjugated goat anti-rabbit IgG (1:1000 dilution; A0545, Sigma-Aldrich, Milan, Italy) or anti-mouse IgG (1:5000 dilution; A9044, Sigma-Aldrich, Milan, Italy) prepared in TTBS containing 1% milk. Membranes were subsequently washed twice with TTBS and once with TBS (20 mM Tris/HCl, pH 7.5, 100 mM NaCl). Protein bands were visualised using a ChemiDoc™ XRS+ System (Bio-Rad Laboratories, Inc., Hercules, CA, USA).

### 2.9. Lipid Extraction

To evaluate any alteration of the lipid profile in Caco-2, cells were seeded in Petri dishes (5 ×10^4^ cells/mL) and, after differentiation, treated for 3 h with fructose (50 mM). Following fructose exposure, the medium was discarded and cells were recovered with PBS containing MgCl_2_ and CaCl_2_. Samples were then centrifuged at 12,500× *g* for 7 min at 4 °C. Lipids were extracted from the resulting pellets according to a modified Folch procedure [33]. For each sample, 700 µL of methanol/chloroform (2:1, *v*/*v*) containing EquiSPLASH™ LIPIDOMIX^®^ Internal Standard Mix (Avanti Polar Lipids, Alabaster, AL, USA) were added. Samples were vortexed at 15-min intervals during a 1-h extraction period, and phase separation was then induced by adding 350 µL chloroform and 150 µL methanol. Following centrifugation at 17,700× *g* for 10 min, 400 µL of the lower organic phase was transferred into glass vials and evaporated to dryness under a gentle stream of nitrogen. The dried lipid extracts were reconstituted in 20 µL of methanol:chloroform (2:1, *v/v*) and 380 µL of isopropanol:acetonitrile:water (2:1:1, *v/v/v*) prior to UHPLC–QTOF–MS analysis in both positive and negative electrospray ionization modes.

### 2.10. LC-MS Lipidomics

Lipidomic analyses were carried out on an Agilent 1290 Infinity UHPLC (Agilent Technologies, Palo Alto, CA, USA) coupled to an Agilent 6540 quadrupole time-of-flight mass spectrometer fitted with a dual ESI source. Separation was performed using a Kinetex C18 column (50 × 2.1 mm, 1.6 µm; Phenomenex, Bologna, Italy), maintained at 50 °C with a flow rate of 0.4 mL min^−1^. In ESI+ mode, mobile phase A contained 60% water and 40% acetonitrile with 10 mM ammonium formate, whereas mobile phase B comprised 90% isopropanol and 10% acetonitrile with 10 mM ammonium formate. For ESI− measurements, the solvent proportions were unchanged, while 10 mM ammonium acetate was used instead of ammonium formate. The gradient started at 60% A and was reduced linearly to 50% over 2.1 min and to 30% over the following 10 min. A was subsequently lowered to 1% and maintained for 1.9 min before returning to the starting composition over 1 min. MS acquisition was performed in both ionization modes. Source settings were: gas temperature, 250 °C; drying gas, 5 L min^−1^; nebulizer pressure, 20 psig; sheath gas temperature, 275 °C; sheath gas flow, 12 L min^−1^; capillary voltage, 4000 V (ESI+) and 3000 V (ESI−); nozzle voltage, 500 V; fragmentor voltage, 400 V; skimmer voltage, 65 V; octopole RF, 750 V. Prior to sample acquisition, external calibration was performed with the Agilent ESI-L Low Concentration Tuning Mix. Continuous mass correction was maintained throughout the analytical run by infusion of the Agilent reference mass solution. Acquisition was controlled with Agilent MassHunter LC/MS Acquisition software, version B.09.00.

### 2.11. mRNA Expression Analysis

CERS2 and CERS6 transcript levels were assessed in differentiated Caco-2 cells cultured in 6-well plates (5 × 10^4^/mL) and exposed to 25 or 50 mM fructose for 1 or 3 h. Following treatment, the fructose-containing medium was removed, and the cells were washed twice with 1× PBS. Total RNA was isolated using the RNeasy Mini Kit^®^ (Qiagen, Hilden, Germany) according to the manufacturer’s instructions. RNA samples were stored at −80 °C until RT-qPCR analysis. SYBR^®^ Green real-time PCR was performed using the TaqPath™ 1-Step RT-qPCR Master Mix (Life Technologies, Waltham, MA, USA). Each reaction was prepared in a final volume of 20 µL and contained 5 µL master mix, 2 µL SYBR^®^ Green solution, 1 µL of each primer, 5 µL RNA extract and 7 µL nuclease-free water. Amplification was performed with a CFX96™ system (Bio-Rad, Hercules, CA, USA) using the following program: 25 °C for 2 min for UNG incubation, 50 °C for 15 min for reverse transcription, followed by 40 cycles of 95 °C for 3 s, 60 °C for 30 s and 81 °C for 2 s. Fluorescence acquisition was performed at the end of each 81 °C step. Three independent biological replicates were analysed for each condition, and results are reported as mean ± SD.

#### Primer Design

Primer pairs targeting CERS2 and CERS6 were designed using the corresponding GenBank accession numbers: NM_022075.1 (CERS2) and NM_203463.3 (CERS6). β-actin was used as the housekeeping reference gene (GenBank accession M10277.1). Oligo version 6 (MedProbe, Oslo, Norway) was used to evaluate potential primer-dimer formation, self-complementarity and primer annealing temperatures (MedProbe, Oslo, Norway). Amplicon melting temperatures (Tms) were estimated with the DINAMelt DNA-hybridisation prediction algorithm [34], using the following parameters: (i) monovalent cation concentration at 0.05 mol/L, (ii) Mg^2+^ at 0.004 mol/L, (iii) a concentration of PCR products (Top/Bottom strands) at 10^−7^ mol/L and (iv) hybridisation temperature at 37 °C [35]. The primer sequences used for amplification were as follows (5′-3′): CERS2-F TAGAGCTTTTGTCCCGGCAG; CERS2-R AATGCTGAAGAGCAGGGACC; CERS6-F GAGGCTGCCAAAATGGCAAA; CERS6-R CATCCTTGGACACCTTGCCT; ACTB-F CACTGTGCCCATCTACGAGG; ACTB-R CCAGGAAGGAAGGCTGGAAG. Relative transcript abundance was calculated using the 2^−ΔΔCt^ [36], with β-actin (ACTB) as the reference gene.

### 2.12. Statistical Analyses

**Figure 1:**
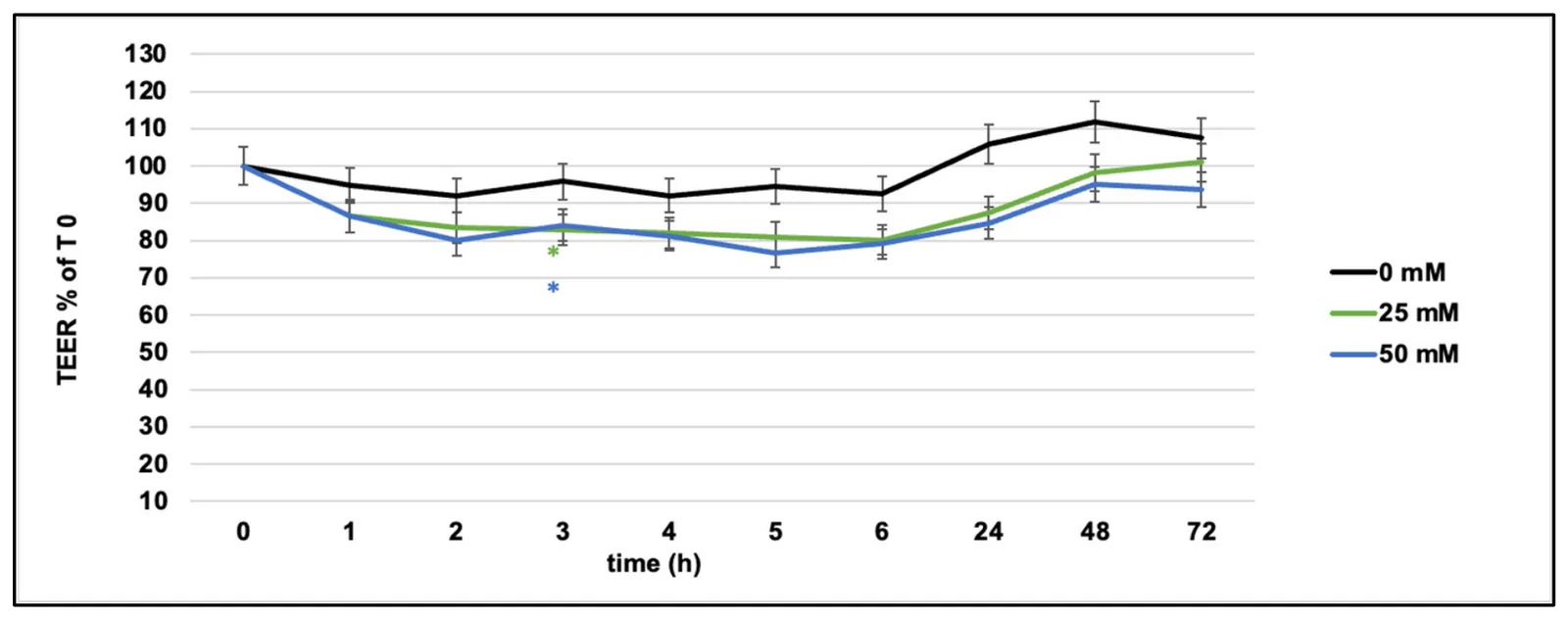
TEER values in Caco-2 cell monolayers incubated for 72 h with fructose at 25 mM and 50 mM. Values are reported as percentage of TEER with respect to 100% of the value at time 0 for each replicate. Data are expressed as mean ± SD (*n* = 3 independent experiments). Statistical analysis was performed by two-way repeated-measures ANOVA (treatment × time, Greenhouse-Geisser corrected) followed by Bonferroni-corrected post hoc comparisons against each group’s own time 0 value. * = *p* < 0.05 vs. time 0.

**Figure 2:**
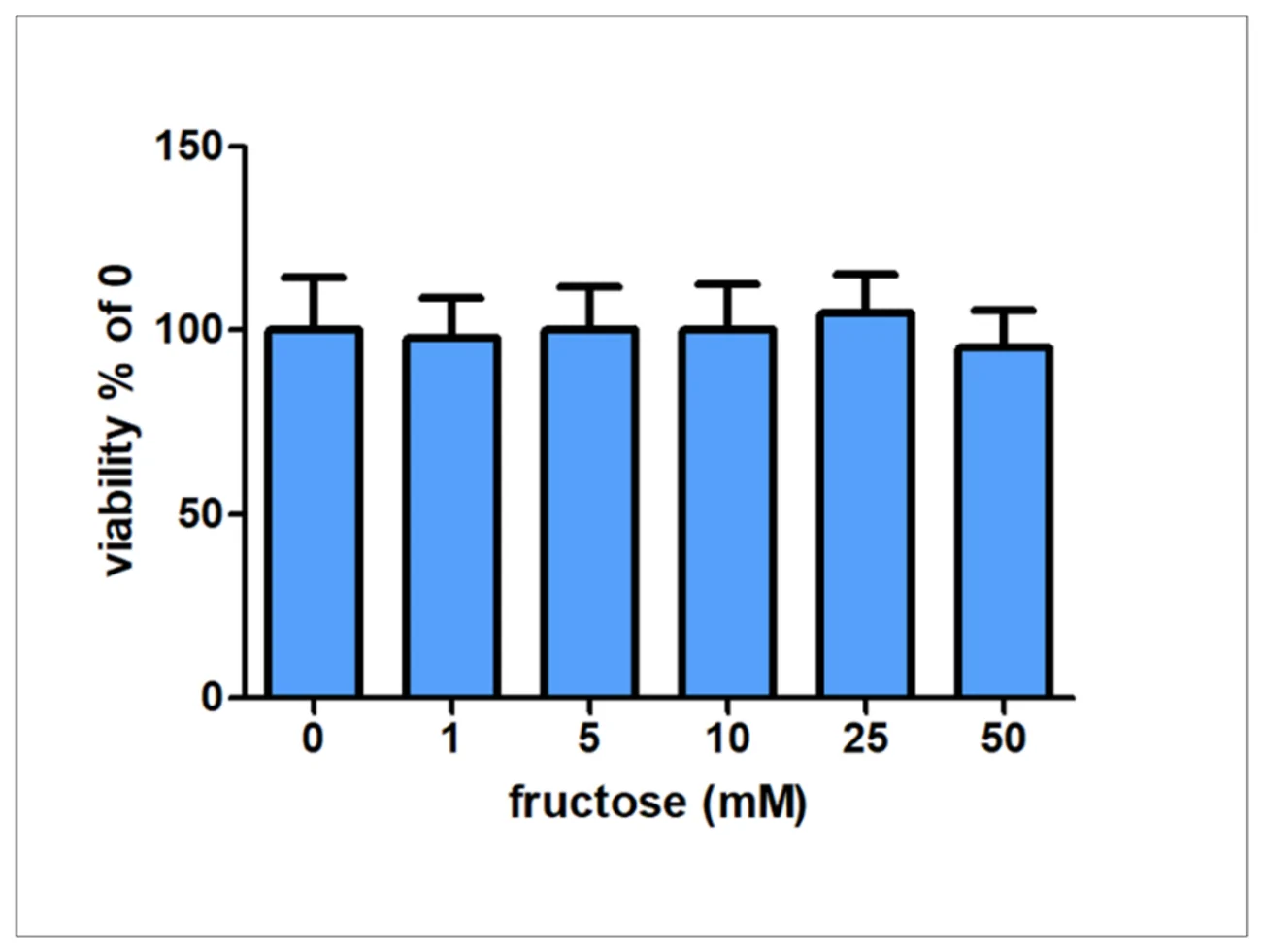
Percentage of viability of Caco-2 cells compared to control (100%/0 mM), incubated for 24 h with different concentrations of fructose (1–50 mM). Data are expressed as mean ± SD (*n* = 16, pooled from 2 independent experiments, 8 wells/experiment).

**Figure 3:**
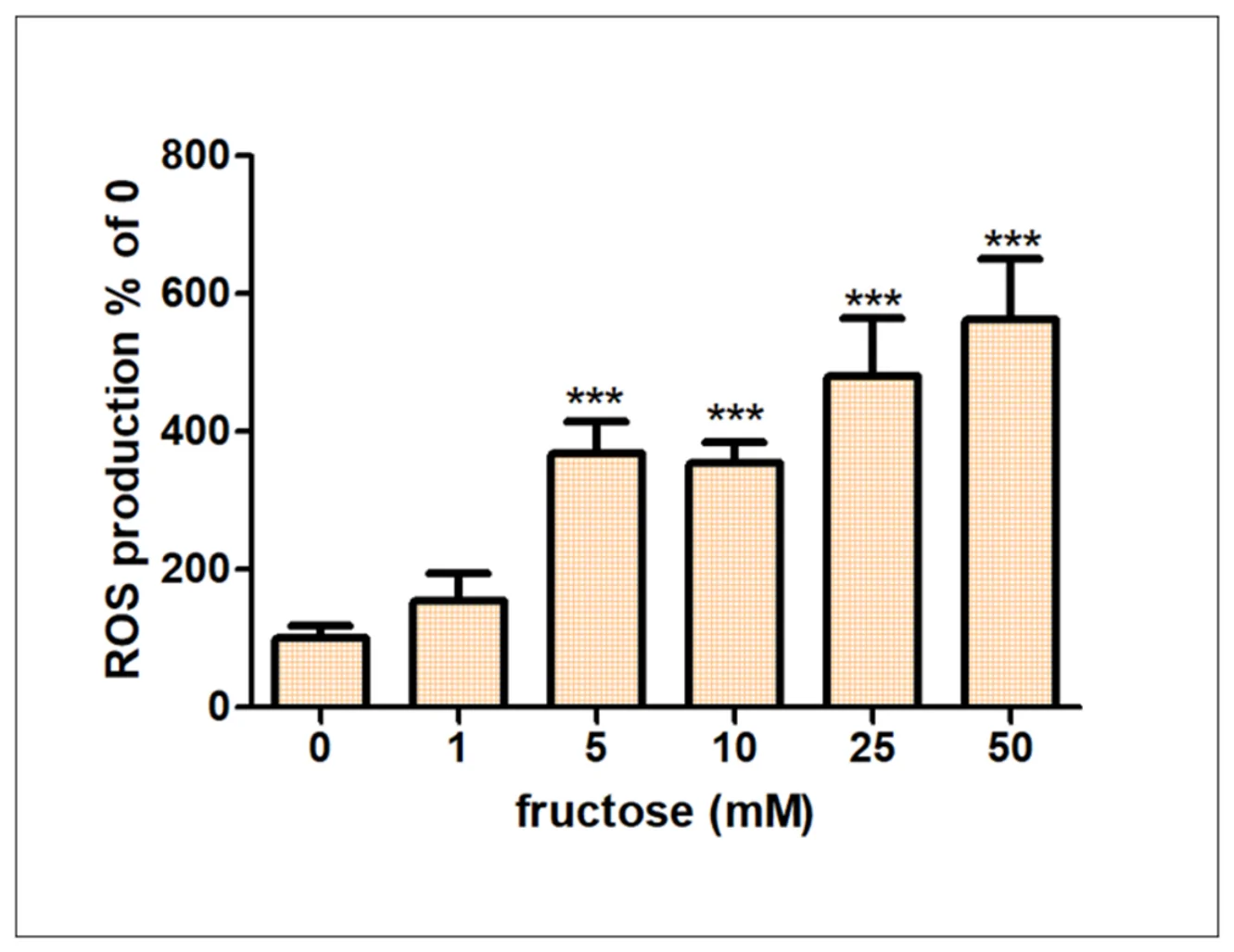
Percentage of ROS compared to the control group (100%/0 mM) at different fructose concentrations of 1–50 mM tested in the Caco-2 cell line. Data are expressed as mean ± SD. *** = *p* < 0.001 vs. 0 mM (*n* = 12, pooled from 2 independent experiments, 6 wells/experiment).

**Figure 4:**
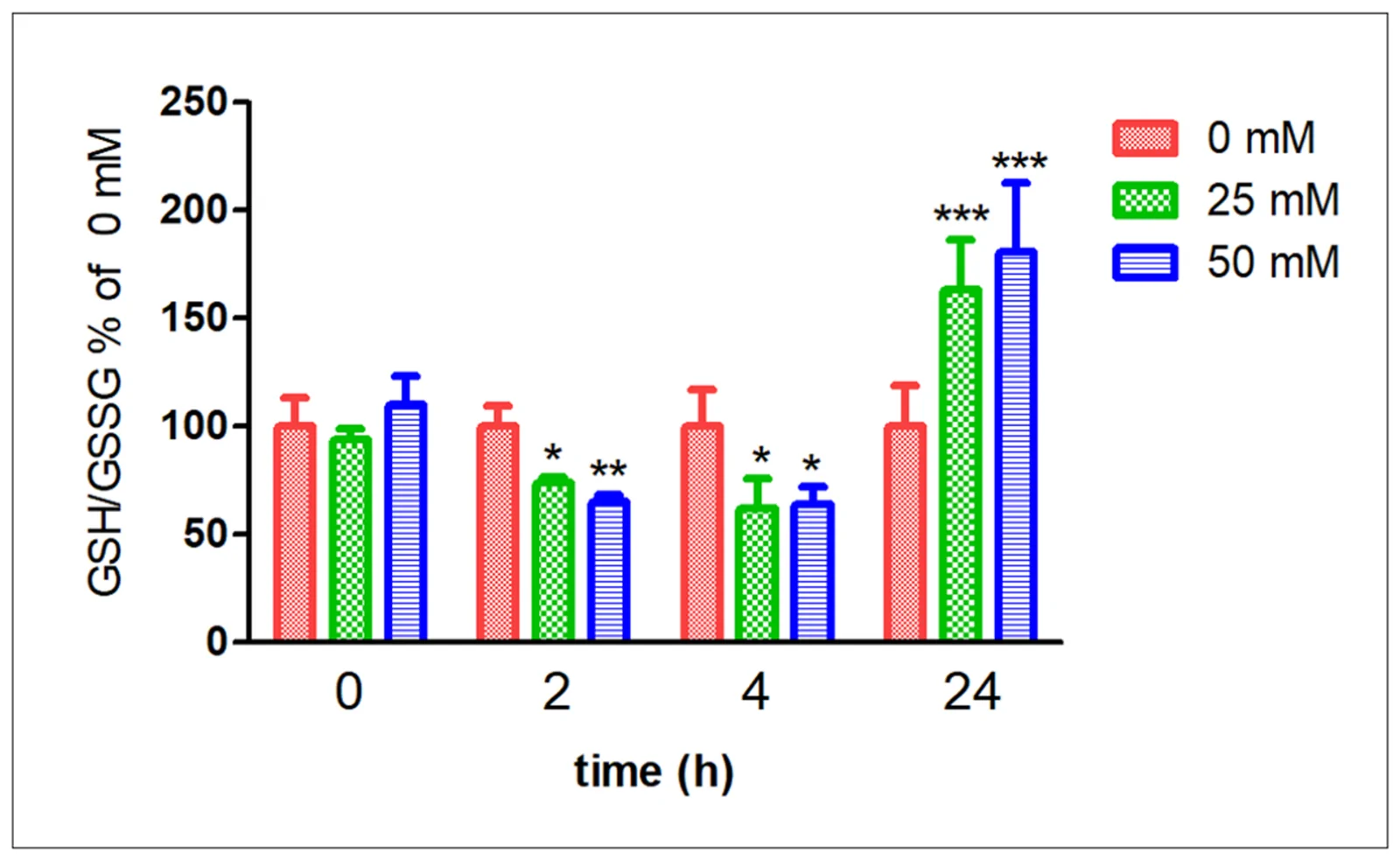
Effect of fructose on intracellular levels of GSH/GSSG ratio at different concentrations (25 and 50 mM) at incubation times of 0, 2, 4 and 24 h compared to the control group (0 mM). Data are expressed as mean ± SD. * = *p* < 0.05, ** = *p* < 0.01, *** = *p* < 0.001 vs. 0 mM (*n* = 6, pooled from 2 independent experiments, 3 wells/experiment).

**Figure 5:**
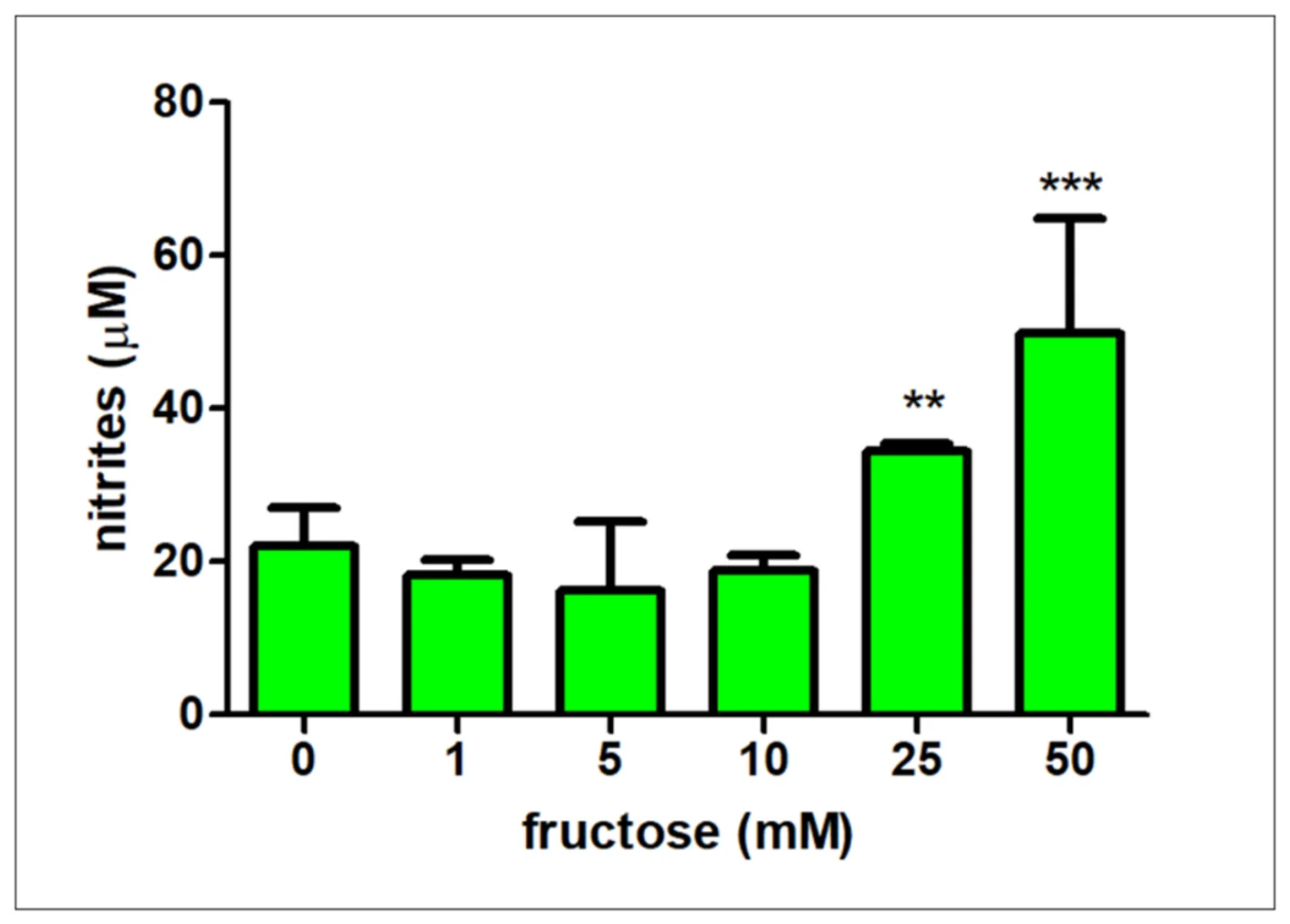
Nitrite values (μmol/L) in Caco-2 cells following treatment with fructose at concentrations of 1–50 mM compared to the control group (0 mM). Data are expressed as mean ± SD. ** = *p* < 0.01, *** = *p* < 0.001 vs. 0 mM (*n* = 12, pooled from 3 independent experiments, 4 wells/experiment).

**Figure 6:**
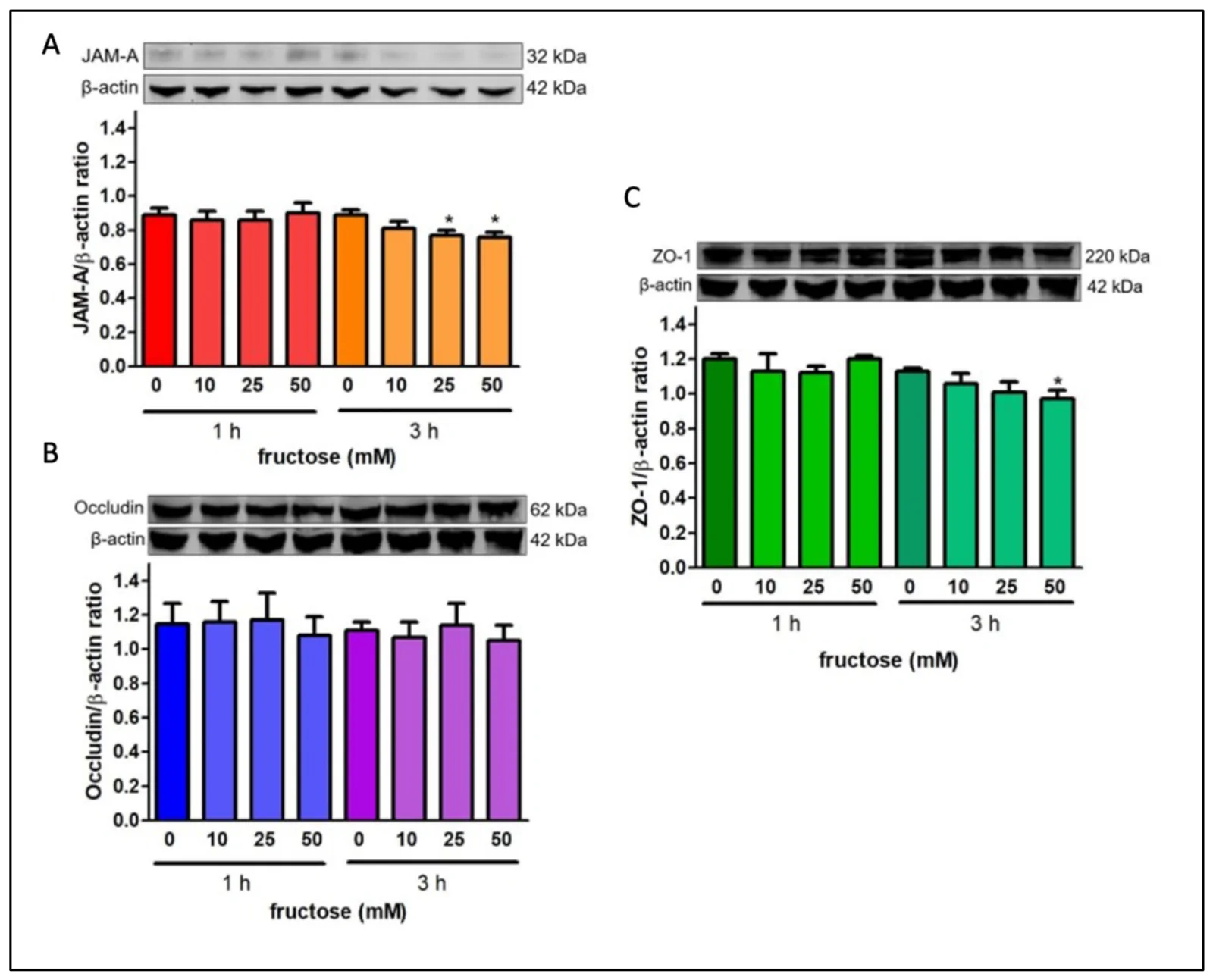
JAM-A (**A**), occludin (**B**), and ZO-1 (**C**)/β-actin ratio measured in differentiated Caco-2 cells treated with fructose (10–50 mM) for 1 and 3 h. * = *p* < 0.05 vs. 0 mM of the same incubation time; (*n* = 3 independent experiments). Representative WB images of the experiment are shown.

**Figure 7:**
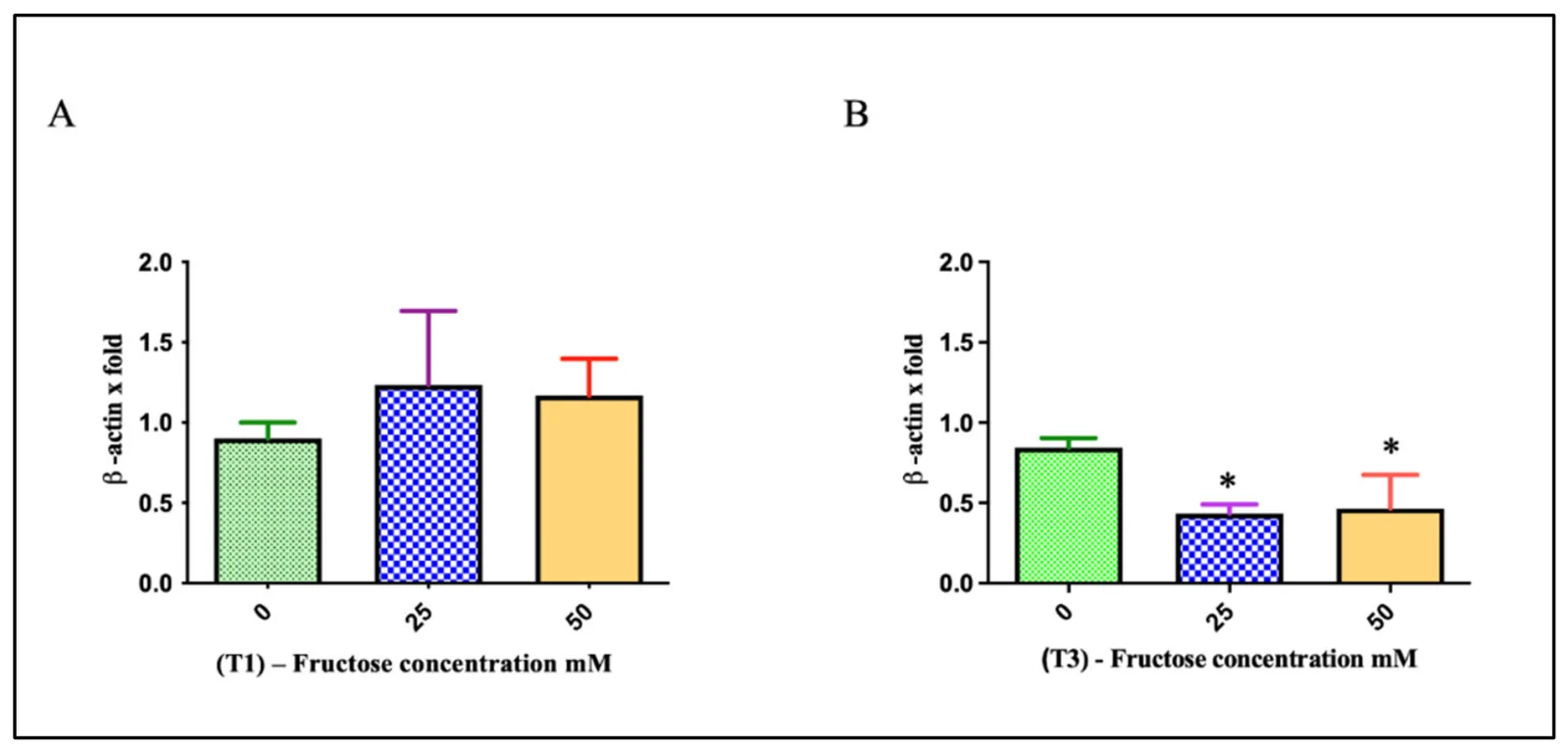
CERS2 mRNA expression after 1 h (**A**) and 3 h (**B**) of fructose treatment. * = *p* < 0.05 vs. 0 mM (*n* = 3 independent experiments).

Statistical analysis was performed using GraphPad Prism 8.1 (GraphPad Software; San Diego, CA, USA). Data are expressed as mean ± standard deviation (SD). For the TEER time-course (Figure 1), percentage values (normalised to each replicate’s own time 0) were analysed by two-way repeated-measures ANOVA (treatment × time), with the Greenhouse-Geisser correction applied given a significant departure from sphericity; Bonferroni-corrected post hoc comparisons were performed against each group’s own time 0 value. Cell viability (MTT, Figure 2), ROS production (Figure 3) and nitrite levels (Figure 5) were each assessed in 2–3 independent experiments, with multiple technical replicate wells per experiment (8, 6 and 4 wells/experiment, respectively), for a total *n* of 16, 12 and 12 wells, respectively, as reported in the corresponding figure legends. GSH/ GSSG levels (Figure 4) were assessed in 2 independent experiments with 3 wells/experiment (total *n* = 6). Western blot (Figures 6 and 7A,B) and qPCR (Figure 7) data derive from *n* = 3 independent experiments. For all these experiments, one-way ANOVA followed by Bonferroni post hoc test was used. For lipidomic data, *p*-values were adjusted for multiple comparisons using the Benjamini–Hochberg false discovery rate (FDR) procedure. An FDR-adjusted *p*-value (q-value) < 0.05 was considered statistically significant. Fold-change values were used to describe the magnitude and direction of the observed changes and were not considered a criterion of statistical significance.

## 3. Results

### 3.1. Measurement of Transepithelial Electrical Resistance

To evaluate the alteration of epithelial barrier integrity following treatment with fructose, TEER was measured in Caco-2 cell monolayers grown in Transwell plates, from time 0 h to 72 h (Figure 1). Two-way repeated-measures ANOVA showed a significant effect of both treatment and time on TEER, with no significant treatment × time interaction (*p* = 0.87). Cells treated with 25 mM and 50 mM fructose showed a consistent downward trend in TEER, reaching approximately 20% below control values across the first 6 h of incubation; after correction for multiple comparisons, this decrease reached statistical significance at the 3 h time point for both the 25 mM (*p* = 0.046) and 50 mM (*p* = 0.043) groups. TEER values subsequently recovered, returning to levels not significantly different from time 0 at 24, 48 and 72 h.

### 3.2. Measurement of Cell Viability (MTT Assay)

To assess potential cytotoxicity, Caco-2 cells were exposed to increasing fructose concentrations (1–50 mM) for 24 h before MTT analysis. As shown in Figure 2, cell viability was not significantly altered by any of the tested fructose concentrations.

### 3.3. ROS Production

To ascertain the possible oxidative stress induced by fructose, the H_2_-DCF-DA test was performed. Caco-2 cells, seeded in 96-well plates, were first treated with H_2_-DCF-DA and subsequently incubated for 1 h at increasing concentrations of fructose (1–50 mM). As shown in Figure 3, fructose increased ROS production starting from the lowest fructose concentration used (1 mM), compared to the control group (100%). From the concentration of 5 mM, the increase became statistically significant, with ROS levels rising by over 500% at the maximum concentration of 50 mM.

### 3.4. Intracellular Content of Reduced and Oxidized Glutathione (GSH/GSSG)

The pro-oxidant response to fructose was further supported by the reduction in intracellular GSH. Treatment of Caco-2 cells with 25 and 50 mM fructose concentrations over various exposure times (0, 2, 4, and 24 h) yielded two distinct outcomes (Figure 4). Short-term incubation (2 and 4 h) resulted in a significant decrease in the GSH/GSSG ratio compared to the untreated control (0 h, 0 mM) at both concentrations. Conversely, prolonged exposure (24 h) resulted in a significant increase in GSH levels at both fructose concentrations tested.

### 3.5. Nitrites Release

Caco-2 cells were treated with fructose at concentrations of 1–50 mM and the nitrite accumulation (μmol/L), as an indirect index of NO production, was determined through the Griess reagent assay. In the cells treated with 25 mM of fructose, a significant increase in nitrite concentrations was observed compared to the control group (0 mM), but an even more significant increase in nitrite concentration was observed in the treatment with 50 mM of fructose, reaching a concentration of 50 μmol/L, more than double compared to the control group (Figure 5).

### 3.6. Effect of Fructose on TJ Protein Level

We next examined whether fructose treatment altered intestinal barrier-associated proteins by analysing JAM-A, occludin and ZO-1 levels by Western blot in Caco-2 cells treated with increasing fructose concentrations (10, 25, and 50 mM) for 1 h or 3 h. JAM-A was not significantly affected by fructose treatment at 1 h at any of the tested concentrations. However, after 3 h of exposure, JAM-A levels were significantly reduced at 25 and 50 mM fructose compared with untreated controls (*p* < 0.05), indicating a time- and dose-dependent effect (Figure 6A). Occludin remained unchanged following fructose exposure, regardless of concentration or treatment duration, with no statistically significant differences observed compared with controls at either 1 or 3 h (Figure 6B). ZO-1 level was not significantly modified after 1 h of fructose treatment. At 3 h, a significant decrease in ZO-1 was observed only at the highest concentration tested (50 mM) compared with the respective control (*p* < 0.05) (Figure 6C). Overall, these results indicate that fructose exposure selectively affects specific TJ proteins in a time- and concentration-dependent manner, with JAM-A and ZO-1 being reduced at longer exposure times and higher concentrations, while occludin amount appears unaffected under the tested experimental conditions.

### 3.7. Expression Patterns of the CERS2 and CERS6 Genes

To investigate the expression of the ceramide synthase genes CERS2 and CERS6, specific primer pairs were designed and used for gene amplification by real-time PCR. Although no variation in CERS6 expression was observed in relation to fructose concentration at T1 and T3 (expression pattern ≃ 1), variations in expression levels were measured for the CERS2 gene. By considering CERS2 mRNA, a significant decrease in gene expression (compared with β-actin mRNA) was observed after 3 h of 25 and 50 mM fructose treatment (*p* < 0.05) (Figure 7).

### 3.8. Untargeted Lipidomics

Untargeted UHPLC-QTOF-MS lipidomics was performed to characterise the lipidomic response of Caco-2 cells following 3 h of fructose exposure. Multivariate analysis (PLS-DA/OPLS-DA) followed by variable importance in projection (VIP) -based feature selection identified sphingolipids as the lipid class most consistently altered between fructose-treated and control cells. As shown in Table 1, 11 ceramides and 6 sphingomyelins were identified among the discriminant lipid species between fructose-treated and control Caco-2 cells, all of which remained statistically significant after Benjamini–Hochberg FDR correction (q < 0.01). Specifically, all discriminant ceramides exhibited increased abundance following fructose treatment, whereas all discriminant sphingomyelins were decreased. The upregulated ceramides included both saturated and monounsaturated molecular species spanning Cer d32:0 to Cer d42:2 and were predominantly characterized by long- and very-long-chain fatty acids, particularly C18:0, C22:0, C24:0 and C24:1. Similarly, the downregulated sphingomyelins were enriched in C20:0, C23:0, C24:0 and C24:1 acyl chains. Additional alterations were also observed in phosphatidylcholines (PC), phosphatidylethanolamines (PE), phosphatidylserines (PS) and triglycerides (TG). However, these lipid classes exhibited heterogeneous regulation, with both increased and decreased molecular species, and are reported in Supplementary Table S1.

## 4. Discussion

High consumption of simple sugars, particularly fructose from added sugars and high-fructose corn syrup is considered a key dietary factor in the development of metabolic diseases and intestinal disorders [5]. Although human evidence is still limited, multiple animal studies consistently show that high-fructose diets worsen colitis severity, essentially via microbiota disruption [37,38,39] and immune activation [40,41,42]. Fructose detrimental effect has also been associated with a direct effect on colonic barrier function in rats, by down-regulating expression of TJ proteins (ZO-1 and occludin) and mucus layer biomarkers (MUC2 and TFF3) [43]. It should be noted that the effects of fructose on intestinal barrier integrity reported in the literature are not entirely consistent, and appear to depend markedly on fructose concentration, exposure duration, and the experimental model used. In an in vivo mouse model receiving physiological, rather than supraphysiological, luminal fructose loads, Zhang et al. found that fructose did not increase paracellular permeability in the small intestine, in contrast to glucose, suggesting that fructose-induced barrier dysfunction may only become apparent above a concentration or dose threshold [44]. Similarly, in a weaned-piglet model more anatomically comparable to humans than rodents, 35-day fructose supplementation reduced ileal TJ proteins gene expression without a measurable inflammatory or oxidative response, indicating that barrier and redox/inflammatory readouts may become uncoupled depending on exposure duration and tissue context [45]. Conversely, other in vivo studies using sustained high-fructose diets have reported clear reductions in occludin and exacerbation of colitis severity, closer in direction to the present findings [46]. These discrepancies are consistent with a model in which fructose-induced barrier dysfunction is concentration- and time-dependent, and possibly transient, as also suggested by the recovery of TEER we observed at later time points; short-term, high-concentration in vitro exposures such as those shown here may capture an early, reversible phase of the response that differs from the barrier phenotype observed after chronic, lower-dose in vivo exposure. Differences in cell/animal model, fructose concentration range, and the specific barrier readout used (TEER vs. paracellular tracer flux assay vs. TJ gene/protein expression) should therefore be considered when comparing across studies. Herein, we aimed to explore candidate mechanisms potentially linking fructose exposure to intestinal epithelial barrier dysfunction, with particular emphasis on oxidative stress, TJ integrity and sphingolipid remodelling in differentiated Caco-2 cells. The tested fructose concentrations (1–50 mM) were selected to reflect the range of fructose levels to which the intestinal epithelium is physiologically and nutritionally exposed following the consumption of fructose-containing foods and beverages. The lower concentrations approximate the Michaelis constant of the intestinal fructose transporter GLUT5 (Km ≈ 6 mM), which mediates fructose uptake across the enterocyte brush-border membrane [47]. Higher concentrations were selected on the basis of reported postprandial luminal sugar concentrations, which can reach approximately 50 mM in the proximal small intestine after ingestion of a carbohydrate-rich meal [48], and of the fructose content of common dietary sources, including sugar-sweetened beverages and fruit juices, in which fructose can reach concentrations of up to 67 g/L in some commercially available 100% fruit juices [49]. These concentrations are also consistent with those previously used to investigate fructose-induced effects on intestinal epithelial permeability and metabolism in Caco-2 monolayers [44,50]. Building on this rationale, the concentration range used for the mechanistic experiments (TEER, Western blot, lipidomics, and CERS2/CERS6 expression analysis) was narrowed relative to the full screening range (1–50 mM) applied for viability and ROS assays. This choice was informed by the concentration-dependence of the redox and nitrosative responses themselves: ROS generation, although detectable from the lowest fructose concentration tested (1 mM), became robustly and significantly elevated only from 5 mM upward, while NO release reached statistical significance exclusively at 25 and 50 mM. Since the working hypothesis of the present study places oxidative and nitrosative stress upstream of TJ alteration and sphingolipid remodelling, the downstream functional and molecular endpoints were therefore assessed at the concentrations shown to reliably engage this stress response, ensuring that any observed effects on barrier integrity and sphingolipid metabolism could be meaningfully related to a measurable oxidative/nitrosative stress response rather than to sub-threshold, likely inconsequential exposures. An osmotic control using an equiosmolar concentration of a non-metabolisable sugar (e.g., mannitol or L-glucose) was not included for the TEER assay or as a control in the other assays; therefore, such controls would be needed to fully dissociate fructose-specific effects from a generic osmotic or carbohydrate-load response and will be included in follow-up studies.

Our experiments in Caco-2 monolayers are consistent with previous studies reporting barrier-impairing effects of fructose; in our in vitro model, fructose exposure led to a reduction in TEER that reached statistical significance at 3 h, consistent with, though not direct proof of, a transient alteration of monolayer permeability. Importantly, fructose did not induce overt cytotoxicity in Caco-2 monolayers, as indicated by the unaltered MTT viability across the whole concentration range tested; the barrier impairment observed therefore reflects a functional, reversible alteration of epithelial integrity rather than cell death. This is in line with the concentration- and time-dependent increase in intracellular ROS production and the parallel, early decrease in the GSH/GSSG ratio, indicating that fructose elicits an oxidative stress response even at non-cytotoxic concentrations. Interestingly, the GSH/GSSG ratio recovered and even exceeded control values after prolonged exposure (24 h), suggesting the activation of adaptive antioxidant mechanisms aimed at restoring cellular redox homeostasis. This adaptive response may also contribute to the concomitant recovery of epithelial barrier integrity, as reflected by the restoration of TEER at later time points [51]. In parallel, fructose stimulated nitrite accumulation, indicative of NO release, in a concentration-dependent fashion, supporting a pro-inflammatory, nitrosative component alongside the oxidative one. These findings are consistent with previous evidence indicating that fructose promotes intestinal permeability through cytochrome P450-2E1-mediated oxidative and nitrosative stress, with induction of iNOS further amplifying the oxidative burden [8]. NO production was indirectly evaluated after 48 h because the Griess reagent assay quantifies nitrite, a stable oxidation product of NO that accumulates progressively in the culture medium over time. Therefore, unlike ROS generation and barrier integrity, which represent early cellular responses, nitrite determination is commonly performed at longer incubation times to allow reliable detection in the medium. Accordingly, fructose selectively affected the TJ protein pattern, with JAM-A and ZO-1 levels significantly reduced after 3 h of exposure at the highest concentrations tested, whereas occludin remained unchanged under all experimental conditions. This apparent discrepancy may reflect protein-specific sensitivity to oxidative and nitrosative insults rather than a generalised loss of the junctional complex. Notably, oxidative stress has been shown to induce redistribution, rather than degradation, of occludin and ZO-1 from the intercellular junctions to the cytoskeleton-associated fraction, with consequent loss of functional barrier properties despite limited changes in total protein content [9,10]; a similar delocalisation phenomenon, rather than proteolytic degradation, may plausibly underlie the reduction in JAM-A and ZO-1 signal observed here, and could also explain the unchanged occludin levels if this protein is affected primarily through relocalisation rather than turnover. Although protein localisation was not directly assessed in the present study, this interpretation is consistent with the transient nature of the TEER decrease, which is more readily explained by a reversible redistribution of junctional components than by their irreversible degradation.

Untargeted lipidomic profiling demonstrated that fructose exposure also induced an early remodelling of sphingolipid metabolism in differentiated Caco-2 cells, characterised by a coordinated increase in ceramides and a concomitant decrease in sphingomyelins. Even though alterations were also observed in other lipid classes, sphingolipids displayed the most homogeneous pattern of regulation, suggesting that these complex lipids represent one of the earliest lipidomic responses to fructose exposure. Although the present study was not designed to investigate metabolic fluxes, the reciprocal regulation of these two lipid classes is consistent with the close metabolic relationship between ceramides and sphingomyelins, which are continuously interconverted through sphingomyelin synthesis and hydrolysis pathways. The central role of this metabolic axis in intestinal epithelial physiology has recently been highlighted, as sphingolipids are essential structural components of epithelial membranes and regulate barrier integrity, differentiation and inflammatory signalling [52]. Several mechanisms may account for ceramide accumulation following fructose exposure. Experimental studies have demonstrated that oxidative stress promotes ceramide generation through multiple pathways, including activation of de novo ceramide synthesis [20], stimulation of sphingomyelin hydrolysis by SMases [53], and modulation of the salvage pathways [54].

One of the most interesting findings emerging from our lipidomic analysis was the selective enrichment of ceramide species containing long- and particularly very-long-chain fatty acids, including C18:0, C22:0, C24:0 and C24:1, which are predominantly synthesized by CERS2 [55]. Unlike C16-containing ceramides, which are frequently associated with apoptosis, insulin resistance and lipotoxicity [56], C22-C24 ceramides exert distinct structural functions by contributing to membrane organization, lipid raft stability and epithelial barrier homeostasis [57]. The predominance of these molecular species therefore suggests that fructose exposure preferentially affects sphingolipid pools involved in membrane architecture rather than inducing a non-selective increase in total ceramide abundance. The coordinated increase in ceramides together with the concomitant decrease in sphingomyelins suggests that fructose exposure induces an early remodelling of the sphingomyelin-ceramide axis.

Among the potential mechanisms linking oxidative stress to ceramide accumulation, activation of sphingomyelinases (SMases) offers one plausible explanation, as these enzymes hydrolyse sphingomyelin into ceramide within minutes of oxidative or nitrosative stimulation, independently of gene transcription [58,59], and have been repeatedly linked to intestinal inflammation and barrier dysfunction [59,60]. The reciprocal changes we observed between sphingomyelin and ceramide species, together with the rapid oxidative/nitrosative response already documented in our model, are compatible with a contribution of SMase-mediated turnover to the ceramide increase seen at 3 h, although SMase activity was not measured directly and this remains to be tested. We also observed a concomitant decrease in CERS2 mRNA at the same time point, despite the accumulation of CERS2-associated long-chain ceramide species; while this may appear counterintuitive, transcriptional downregulation of ceramide synthases in response to elevated substrate or product flux has been described elsewhere [61,62], and could plausibly represent an early compensatory response that would only affect ceramide pools over a longer timescale than studied here. We offer this combination of SMase-mediated turnover and compensatory CERS2 downregulation as one internally consistent, testable hypothesis for the observed sphingolipid changes, rather than as a demonstrated pathway; direct time-course lipidomics and SMase activity measurements, outlined in the Limitations below, will be required to test it. Beyond its biosynthetic origin, an excess of long- and very-long-chain ceramides could also plausibly alter the biophysical properties of the membrane surrounding TJ complexes, given that such ceramide species are known to increase membrane order and promote rigid, ceramide-enriched domains [14,15]; this offers one possible, untested, lipid-centered complement to the redox-based explanation discussed above for the delocalisation of JAM-A and ZO-1.

This study has some limitations that should be acknowledged. First, lipidomic profiling was performed at a single time point (3 h), which does not allow us to establish the temporal relationship between sphingolipid remodelling and barrier dysfunction, and SMase activity was not measured directly; consequently, the SMase-driven mechanism proposed here, and more broadly the molecular mechanisms linking sphingolipid remodelling, CERS2 regulation and TJ organisation, remain a hypothesis generated by, rather than directly demonstrated in, this dataset. Second, the number of biological replicates for Western blot and qPCR (*n* = 3) is modest and limits statistical power; this was partly mitigated by adjustment for multiple comparisons in the lipidomic dataset but should be considered when interpreting the protein and gene expression findings. Third, barrier function was assessed exclusively by TEER; complementary paracellular tracer flux assays (e.g., FITC-dextran or Lucifer Yellow) would provide more direct evidence of macromolecular permeability. Addressing these points, in particular through time-resolved lipidomic and functional analyses integrating SMase activity, CERS regulation and TJ organization, will be the focus of future studies building on the present exploratory dataset.

## 5. Conclusions

In conclusion, the present exploratory study shows that fructose exposure is associated with an early, measurable impairment of epithelial barrier function in differentiated Caco-2 cells, alongside oxidative and nitrosative stress, altered TJ protein levels, and sphingolipid remodelling. Given the screening nature of the experimental design, these findings should be regarded as hypothesis-generating rather than as establishing a fully characterized causal mechanism. The observed increase in ceramide species, concomitant reduction in sphingomyelins, and downregulation of CERS2 expression suggest that sphingolipid homeostasis is altered during the early response to fructose, although the underlying mechanisms require further investigation. Overall, these findings provide new insights into the molecular events linking fructose exposure to intestinal epithelial dysfunction and identify sphingolipid remodelling as a potential contributor to the deleterious effects of high fructose intake on gut barrier integrity.

## Figures and Tables

**Table 1:** Comparison of lipid species down- and upregulated in Caco-2 cells treated with fructose (50 mM) for 3 h.

Attribution	Adduct	m/z Experimental	RT (Min)	VIP	CTR Mean ± SD	Fructose Mean ± SD	Fold Change	Log2fc	*p*-Value	FDR-Adjusted *p*-Value (q)	Regulation in Treated Samples	Significance
Cer d18:0/16:0	+H+	540.5363	4.41	1.27	3.792 ± 0.687	10.494 ± 1.057	2.768	1.469	4.69 × 10^−8^	1.60 × 10^−7^	+	***
Cer d18:0/24:1	+H+	650.6463	8.68	1.24	1.807 ± 0.388	12.479 ± 1.658	6.908	2.788	1.17 × 10^−6^	1.80 × 10^−6^	+	***
Cer d18:0/14:0	+H+	510.4893	3.29	2.23	196.268 ± 20.385	270.469 ± 18.718	1.378	0.463	1.31 × 10^−5^	1.85 × 10^−5^	+	***
Cer d18:0/14:1	+H+	512.5043	3.56	1.11	61.431 ± 18.571	89.485 ± 13.305	1.457	0.543	0.00786	0.00786	+	**
Cer d18:0/15:0	+H+	526.5203	3.97	1.91	47.231 ± 3.329	74.880 ± 5.073	1.585	0.665	2.00 × 10^−7^	4.85 × 10^−7^	+	***
Cer d18:0/15:1	M+H-H_2_O	506.4933	3.67	1.16	43.364 ± 2.675	66.056 ± 2.325	1.523	0.607	1.25 × 10^−9^	7.10 × 10^−9^	+	***
Cer d18:1/16:0	+H+	538.5213	4.50	1.93	1066.238 ± 414.062	1838.873 ± 417.922	1.725	0.786	0.00459	0.00488	+	**
Cer d18:2/22:0	+H+	620.5976	6.23	1.18	72.879 ± 4.925	91.923 ± 5.513	1.261	0.335	1.98 × 10^−5^	2.60 × 10^−5^	+	***
Cer d18:0/24:0	+H+	652.6623	9.13	1.32	32.818 ± 0.965	58.958 ± 1.814	1.797	0.845	6.66 × 10^−11^	1.13 × 10^−9^	+	***
Cer d18:1/24:0	+H+	650.6463	8.68	1.24	149.585 ± 9.954	330.810 ± 28.702	2.212	1.145	5.58 × 10^−7^	1.05 × 10^−6^	+	***
Cer d18:1/24:1	+H+	648.6303	7.39	1.23	255.746 ± 24.849	430.913 ± 18.215	1.685	0.753	1.10 × 10^−8^	4.68 × 10^−8^	+	***
SM d32:0	+H+	705.5923	3.19	1.44	9.442 ± 0.565	4.844 ± 0.945	0.513	−0.963	7.54 × 10^−7^	1.28 × 10^−6^	−	***
SM d38:1	+H+	759.6373	4.60	1.24	15.886 ± 0.937	5.737 ± 0.976	0.361	−1.469	1.56 × 10^−10^	1.33 × 10^−9^	−	***
SM d41:1	+H+	801.6853	6.21	1.32	9.601 ± 0.851	4.685 ± 0.677	0.488	−1.035	8.25 × 10^−8^	2.34 × 10^−7^	−	***
SM d41:2	+H+	799.6683	5.40	2.71	12.617 ± 1.445	1.668 ± 0.369	0.132	−2.919	3.40 × 10^−7^	7.23 × 10^−7^	−	***
SM d42:0	+H+	817.7153	7.18	1.28	8.997 ± 1.221	5.289 ± 0.516	0.588	−0.766	7.25 × 10^−5^	8.80 × 10^−5^	−	***
SM d42:2	+H+	813.6863	5.60	2.05	9.070 ± 1.375	5.215 ± 0.412	0.575	−0.798	0.00018	0.00021	−	***

Data includes theoretical and experimental m/z values, adduct types, retention times (RT), fatty acid composition and variable importance in projection (VIP) scores related to ceramide (Cer) and sphingomyelins (SM). Six samples per condition (6 vs. 6) were analysed. + = upregulated vs. untreated cells (*p* < 0.05); − = downregulated vs. untreated cells (*p* < 0.05). Fold change = fructose/CTR; *p*-values were adjusted for multiple comparisons using the Benjamini–Hochberg FDR procedure. ** = *p* < 0.01; *** = *p* < 0.001.

## Data Availability

The data are not publicly available because they are part of an ongoing research project; they are available from the corresponding author upon reasonable request.

## Supplementary Materials

The following supporting information can be downloaded at https://www.mdpi.com/article/10.3390/nu18203359/s1, Table S1: Comparison of lipid species upregulated in Caco-2 cells treated with fructose (50 mM) for 3 h.

## Abbreviations

The following abbreviations are used in this manuscript:

ANOVA	Analysis of variance
Caco-2	Human colorectal adenocarcinoma cell line
CERS	Ceramide synthase
CERS2	Ceramide synthase 2
CERS6	Ceramide synthase 6
DMEM	Dulbecco’s Modified Eagle Medium
DMSO	Dimethyl sulfoxide
DNA	Deoxyribonucleic acid
ESI	Electrospray ionization
FBS	Fetal bovine serum
FDR	False discovery rate
GSH	Reduced glutathione
GSSG	Oxidized glutathione
HPLC	High-performance liquid chromatography
HPLC-EC	High-performance liquid chromatography coupled with electrochemical detection
IBD	Inflammatory bowel disease
JAM-A	Junctional adhesion molecule-A
LC-MS	Liquid chromatography–mass spectrometry
MASLD	Metabolic dysfunction-associated steatotic liver disease
MPA	Metaphosphoric acid
MTT	3-(4,5-Dimethylthiazol-2-yl)-2,5-diphenyltetrazolium bromide
NADPH	Nicotinamide adenine dinucleotide phosphate
NAFLD	Non-alcoholic fatty liver disease
NO	Nitric oxide
OPLS-DA	Orthogonal partial least squares discriminant analysis
PBS	Phosphate-buffered saline
PC	Phosphatidylcholine
PCR	Polymerase chain reaction
PE	Phosphatidylethanolamine
PLS-DA	Partial least squares discriminant analysis
PS	Phosphatidylserine
ROS	Reactive oxygen species
RNS	Reactive nitrogen species
RT-qPCR	Reverse transcription quantitative polymerase chain reaction
SD	Standard deviation
S1P	Sphingosine-1-phosphate
SM	Sphingomyelin
TBS	Tris-buffered saline
TEER	Transepithelial electrical resistance
TFA	Trifluoroacetic acid
TG	Triglycerides
TJ	Tight junction
TTBS	Tris-buffered saline with Tween 20
UHPLC	Ultra-high-performance liquid chromatography
UHPLC-QTOF-MS	Ultra-high-performance liquid chromatography coupled with quadrupole time-of-flight mass spectrometry
UNG	Uracil-DNA glycosylase
VIP	Variable importance in projection
ZO-1	Zonula occludens-1
