# Supplementary material for: Fructose Promotes Oxidative Stress, Sphingolipid Remodelling and Transient Intestinal Barrier Dysfunction in Differentiated Caco-2 Cells
Source: Nutrients. 2026 Oct 14;18(20):3359. doi: 10.3390/nu18203359 (PMC13708186; doi:10.3390/nu18203359)
Supplement: Supplementary file 1 [file nutrients-18-03359-s001.zip › nutrients-4583332-supplementary.pdf]

Fructose promotes oxidative stress, sphingolipid remodelling and transient intestinal barrier dysfunction in differentiated Caco-2 cells

Gabriele Serreli[1*], Maria Paola Melis[1], Claudia Guerriero[1], Cristina Manis[2], Giulia Tolle[2], Giuseppina Sanna[1], Germano Orrù[3], Sara Fais[3], Pierluigi Caboni[2], &

Monica Deiana[1]

[1]Department of Biomedical Sciences, University of Cagliari, Monserrato (Cagliari), 09042, Italy
[2]Department of Life and Environmental Sciences, University of Cagliari, Monserrato (Cagliari), 09042, Italy
[3]Department of Surgical Sciences, Oral Biotechnology Laboratory (OBL), University of Cagliari, Cagliari, 09121, Italy
*Correspondence: gabriele.serreli@unica.it; Tel.: +39-0706754185

Supplementary files

Table S1 Comparison of lipid species upregulated in Caco-2 cells treated with fructose (50 mM) for 3 h.

Attribution	Adduct	*m/z experimental*	RT (min)	Fatty acid composition	VIP	Regulation in treated samples
LPC 18:0	$+H^+$	524,3723	1.13	18:0	2.59	+
PC 28:0	$+H^+$	678,5073	2.38	11:0; 17:0	1.31	+
PC 29:0	$+H^+$	692,5253	2.69	19:0; 10:0	1.35	+
PC 29:1	$+H^+$	690,5093	3.19	15:1; 14:0	2.68	+
PC 30:0	$+H^+$	706,5403	3.10	10:0; 20:0	1.17	-
PC 30:1	$+H^+$	688,5283	3.91	16:0; 14:1	1.33	+
PC 31:1	$+H^+$	718,5413	3.90	15:1/16:0	1.31	-
PC 32:1	$+H^+$	732,5573	3.70	15:1; 17:0	1.18	+
PC 32:2	$+H^+$	730,5403	2.54	15:1; 17:1	1.69	+
PC 33:1	$+H^+$	746,5713	3.38	15:1; 18:0	1.12	+
PC 33:2	$+H^+$	744,5573	3.97	15:1; 18:1	1.71	+
PC 34:1	$+H^+$	760,5883	3.78	15:0; 19:1	1.81	+
PC 34:2	$+H^+$	758,5733	3.16	15:1; 19:1	1.18	+
PC 33:2	$+H^+$	744,5573	3.97	15:1; 18:1	1.71	+
PC 35:6	$+H^+$	764,5253	2.89	20:5; 15:1	1.87	-
PC 36:1	$+H^+$	788,6193	4.69	21:0; 15:1	1.34	-
PC 36:2	$+H^+$	786,6033	3.86	18:1; 18:1	1.26	+
PC 37:2	$+H^+$	800,6193	4.27	15:1; 22:1	2.83	-

PC 38:2	$+H^+$	814,6343	4.72	21:0; 17:2	1.70	+
PC 40:2	$+H^+$	842,6563	5.73	22:1; 18:1	1.29	+
PC 42:2	$+H^+$	870,6963	6.86	22:1; 20:1	1.23	-
PE 32:1	$+H^+$	674,5123	3.52	16:0; 16:1	1.28	-
PE 33:0	$+H^+$	692,5573	3.40	18:0; 15:0	2.40	+
PE 33:1	$+H^+$	704,5253	2.46	19:1; 14:0	1.31	+
PE 34:3	$+H^+$	700,5283	3.59	16:0; 18:3	1.81	-
PS 29:0	$+H^+$	680,4813	5.90	16:0; 13:0	1.67	+
PS 41:7	$+H^+$	848,5433	3.16	19:1; 22:6	1.13	+
PS 42:1	$+H^+$	860,6693	1.68	20:0; 22:1	1.56	+
TG 46:0	NH_4^+	796,7403	13.33	14:0; 16:0; 16:0	1.57	-
TG 46:2	NH_4^+	792,7103	11.49	13:0; 13:0; 20:2	1.24	+
TG 47:2	$+H^+$	806,7253	12.30	14:0; 16:1; 17:1	1.19	-
TG 48:0	$+H^+$	793,7673	14.85	16:0; 14:0; 18:0	1.63	+
TG 48:1	NH_4^+	822,7563	13.42	15:0; 16:0; 17:1	1.25	+
TG 48:2	$+H^+$	820,7413	12.54	15:0; 16:0; 17:2	1.22	+
TG 50:4	$+H^+$	827,7113	13.40	14:1; 22:2; 14:1	1.28	+
TG 51:1	NH_4^+	864,7993	13.54	15:0; 22:0; 14:1	1.42	+
TG 51:3	$+H^+$	860,7713	13.12	15:1; 18:1; 18:1	1.30	+
TG 52:1	NH_4^+	878,8153	13.54	14:0; 24:1; 14:0	1.50	+
TG 52:2	NH_4^+	876,8043	14.10	14:1; 18:0; 20:1	1.28	+
TG 53:1	$+H^+$	892,8333	14.19	15:1; 19:0; 19:0	1.40	+
TG 53:6	$+H^+$	865,7263	13.12	15:0; 18:2; 20:4	1.36	-
TG 54:3	NH_4^+	902,8203	14.18	18:0; 18:1; 18:2	1.65	-
TG 54:5	$+H^+$	881,7583	14.14	16:0; 22:5; 16:0	1.88	+
TG 54:7	$+H^+$	885,7323	14.38	18:4; 18:3; 18:0	1.34	+
TG 54:8	$+H^+$	883,7163	13.83	18:4; 18:4; 18:0	1.22	-
TG 55:4	$+H^+$	897,7883	14.84	15:0; 20:4; 20:0	1.39	+
TG 57:6	$+H^+$	921,7893	14.47	20:1; 15:0; 22:5	1.52	+
TG 58:0	NH_4^+	964,9303	15.57	21:0; 18:0; 19:0	2.76	-
TG 58:1	$+H^+$	962,9113	15.84	18:0; 20:0; 20:1	1.50	-
TG 58:3	$+H^+$	958,8813	15.15	17:1; 19:1; 22:1	2.30	-
TG 58:6	$+H^+$	935,8063	14.73	20:0; 18:3; 20:3	1.51	+
TG 59:4	NH_4^+	970,8803	14.23	19:0; 20:0; 20:4	1.91	+
TG 59:6	$+H^+$	949,8213	14.94	15:0; 20:5; 24:1	1.30	+
TG 60:0	NH_4^+	992,9613	15.84	19:0; 21:0; 20:0	2.05	-
TG 60:1	NH_4^+	990,9423	15.54	20:0; 20:1; 20:0	1.45	-

Data includes theoretical and experimental m/z values, adduct types, retention times (RT), fatty acid composition and variable importance in projection (VIP) scores related to lysophosphatidylcholine (LPC), phosphatidylcholines (PC), lysophosphoetanolamine (PE), phosphatidylserine (PS) and triacylglycerols (TG). Six samples per condition (6 vs. 6) were analyzed. + = upregulated vs untreated cells ($p < 0.05$); - = downregulated vs untreated cells ($p < 0.05$).
